# Supplementary material for: Comprehensive chromatin proteomics resolves functional phases of pluripotency and identifies changes in regulatory components
Source: Nucleic Acids Res. 2023 Feb 20;51(6):2671–90. doi: 10.1093/nar/gkad058 (PMC10085704; doi:10.1093/nar/gkad058)
Supplement: gkad058_Supplemental_Files [file gkad058_supplemental_files.zip › Supplementary figures_combined_revisions_final.pdf]

SUPPLEMENTARY FIGURES for:

Comprehensive chromatin proteomics resolves functional phases of pluripotency and identifies changes in regulatory components

Authors:
Enes Ugur, Alexandra de la Porte, Weihua Qin, Sebastian Bultmann, Alina Ivanova, Micha Drukker, Matthias Mann*, Michael Wierer*, Heinrich Leonhardt*

Correspondence:
mmann@biochem.mpg.de, michael.wierer@sund.ku.dk and h.leonhardt@lmu.de

This PDF includes:
Figure S1 (Related to Figure 1)
Figure S2 (Related to Figure 1)
Figure S3 (Related to Figure 2)
Figure S4 (Related to Figure 3)
Figure S5 (Related to Figure 3)
Figure S6 (Related to Figure 3)
Figure S7 (Related to Figure 3)
Figure S8 (Related to Figure 3)
Figure S9 (Related to Figure 3)
Figure S10 (Related to Figure 3)
Figure S11 (Related to Figure 4)
Figure S12 (Related to Figure 6)

Figure S1 (Related to Figure 1)

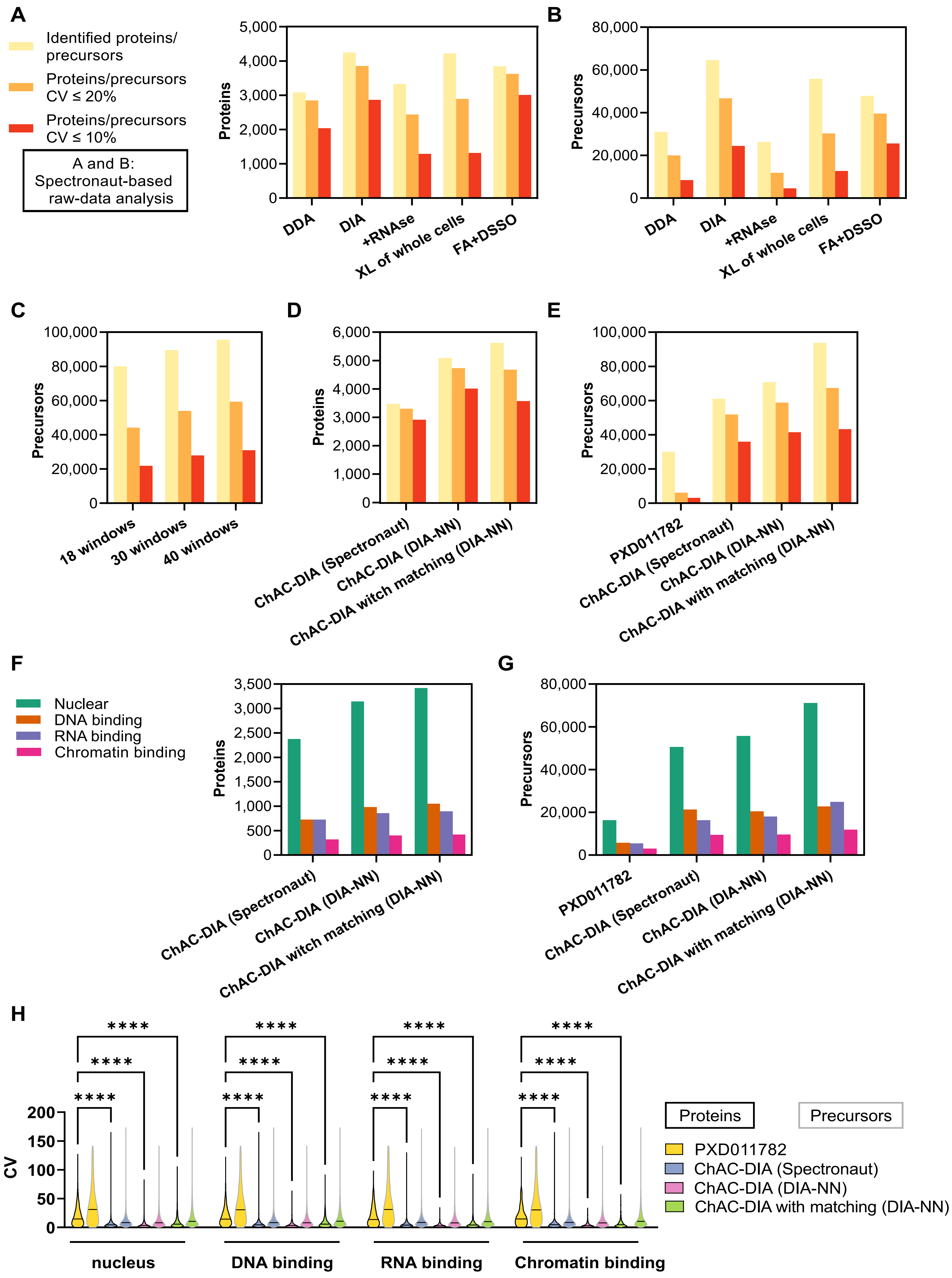

Figure S2 (Related to Figure 1)

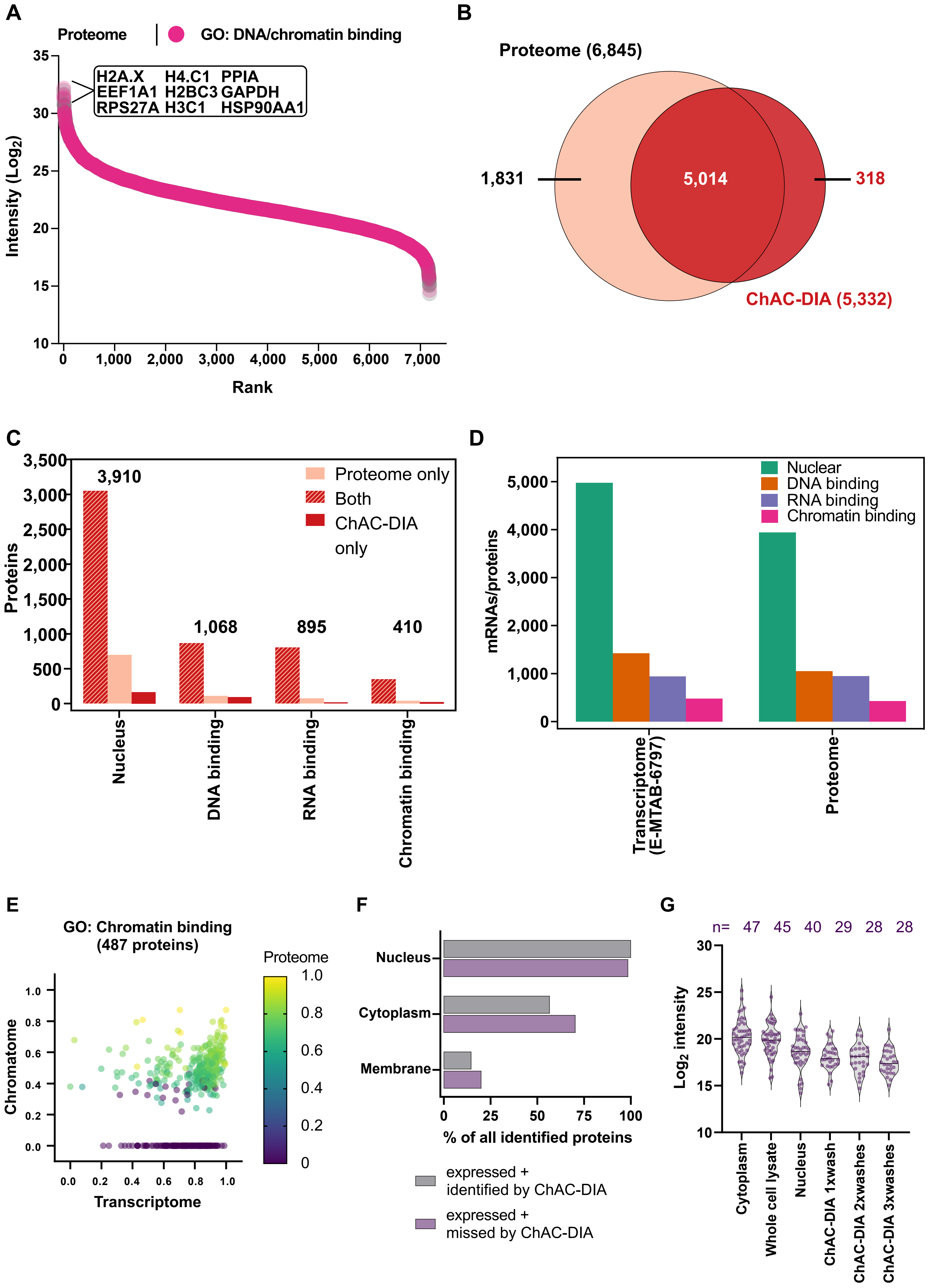

Figure S3 (Related to Figure 2)

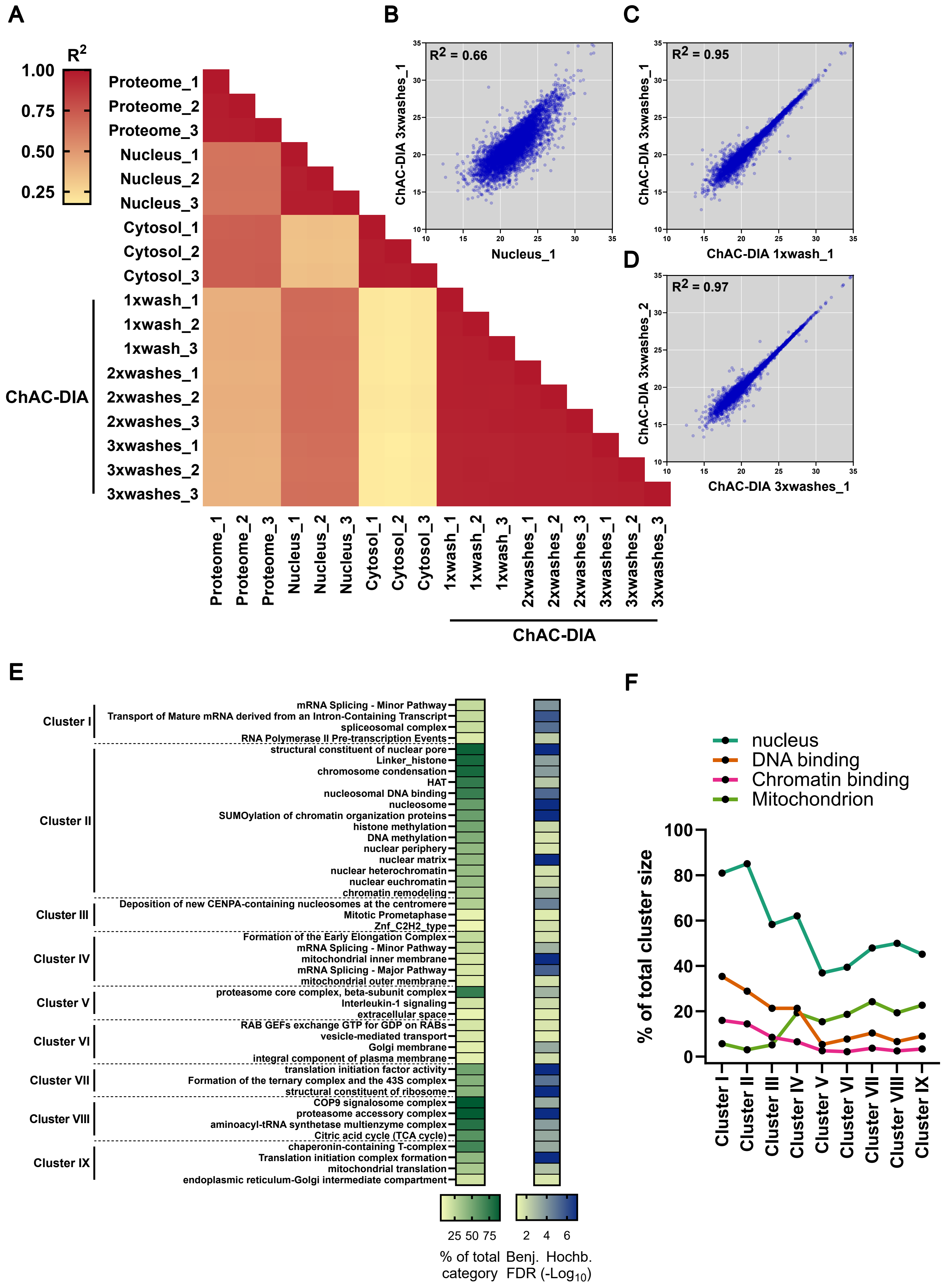

Figure S4 (Related to Figure 3)

Pluripotency/differentiation marker proteins

A) ANOVA significant (FDR < 0.05, fold change difference ≥ 2)

B) Absent in at least one pluripotency phase

C) Not ANOVA significant between any pluripotency phase

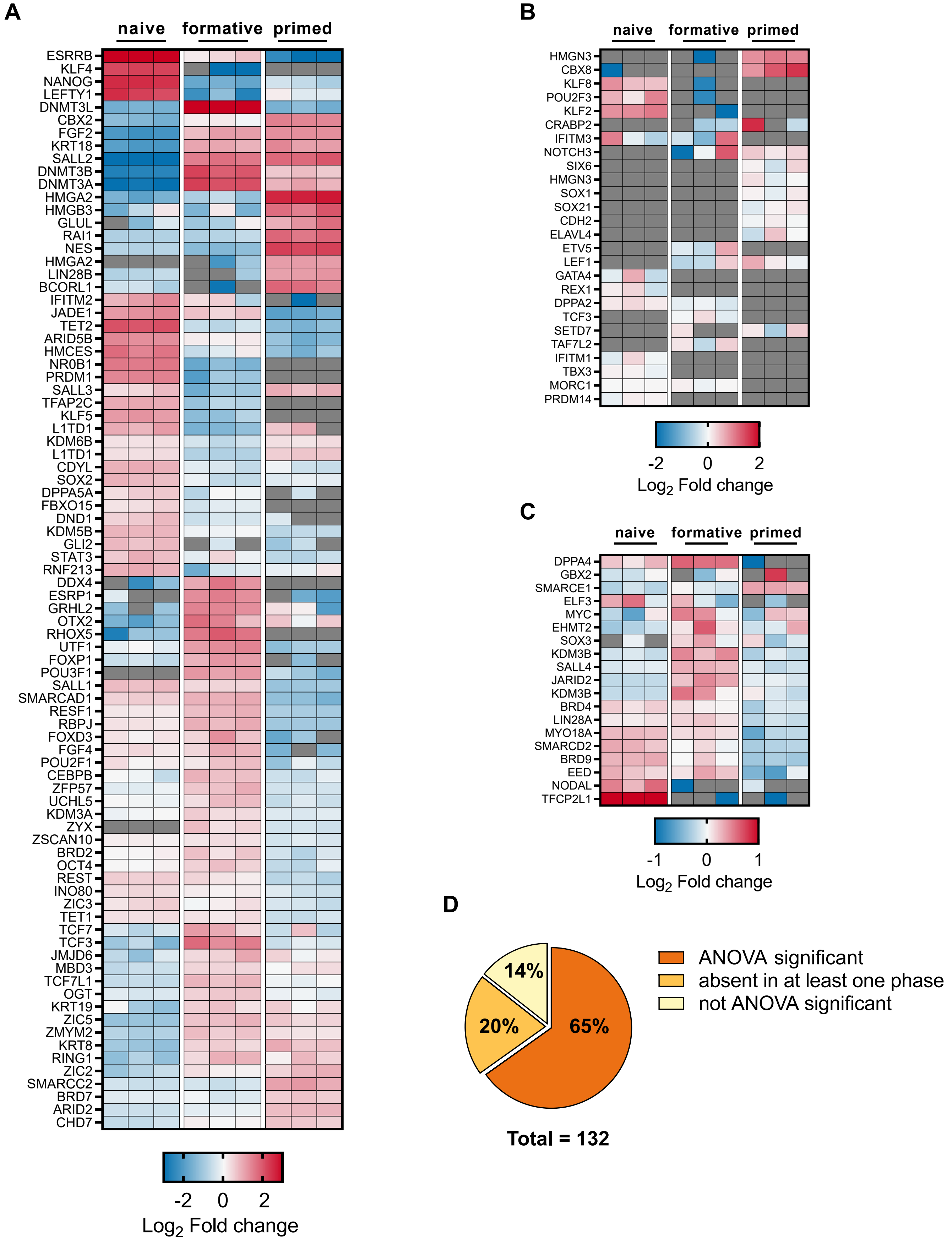

Figure S5 (Related to Figure 3)

GO "Transcription factor activity"

A) ANOVA significant (FDR < 0.05, fold change difference ≥ 2)

B) Absent in at least one pluripotency phase

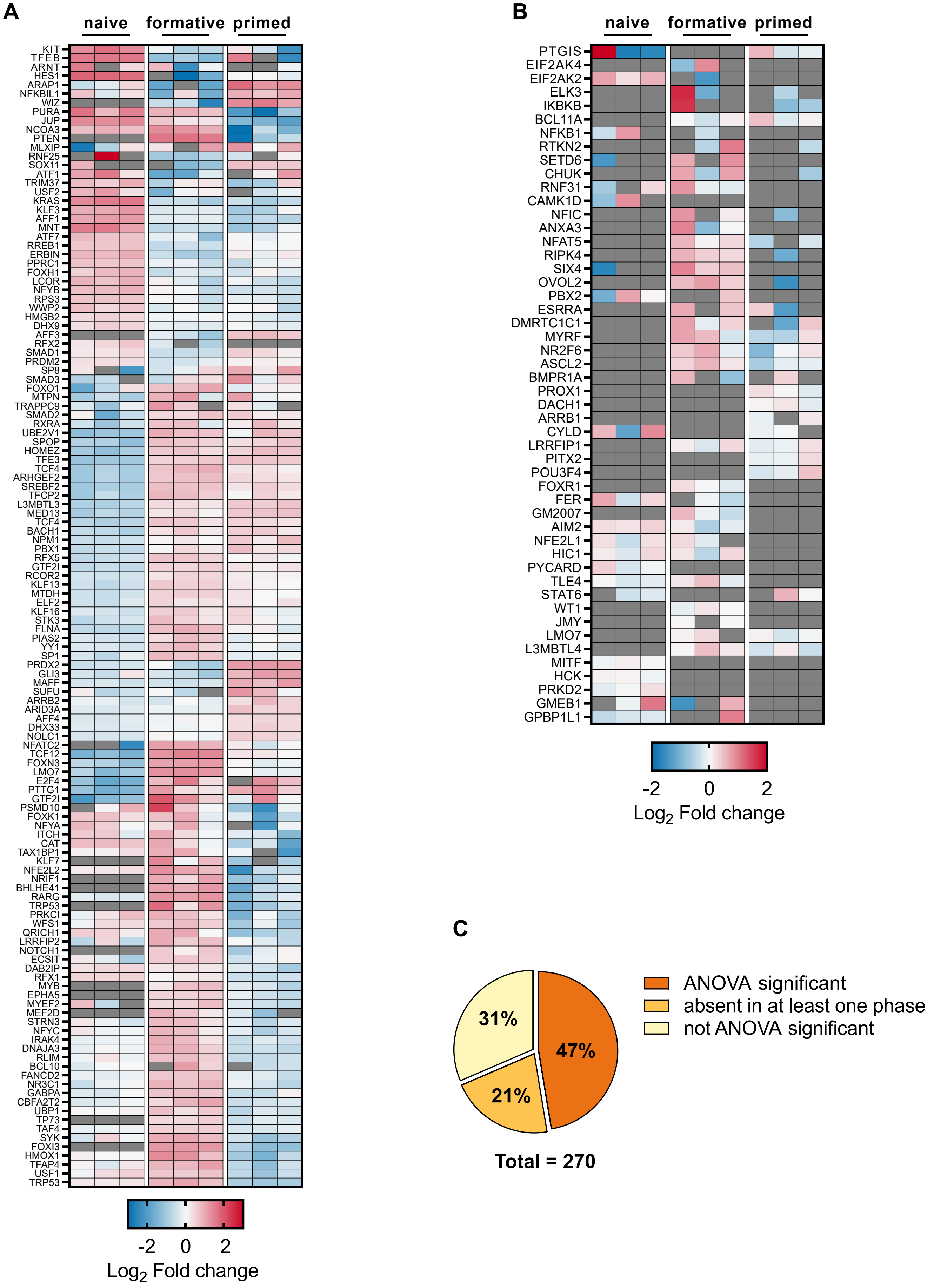

Figure S6 (Related to Figure 3)

Proteins related in epigenetic regulation

A) ANOVA significant (FDR < 0.05, fold change difference ≥ 2)

B) Absent in at least one pluripotency phase

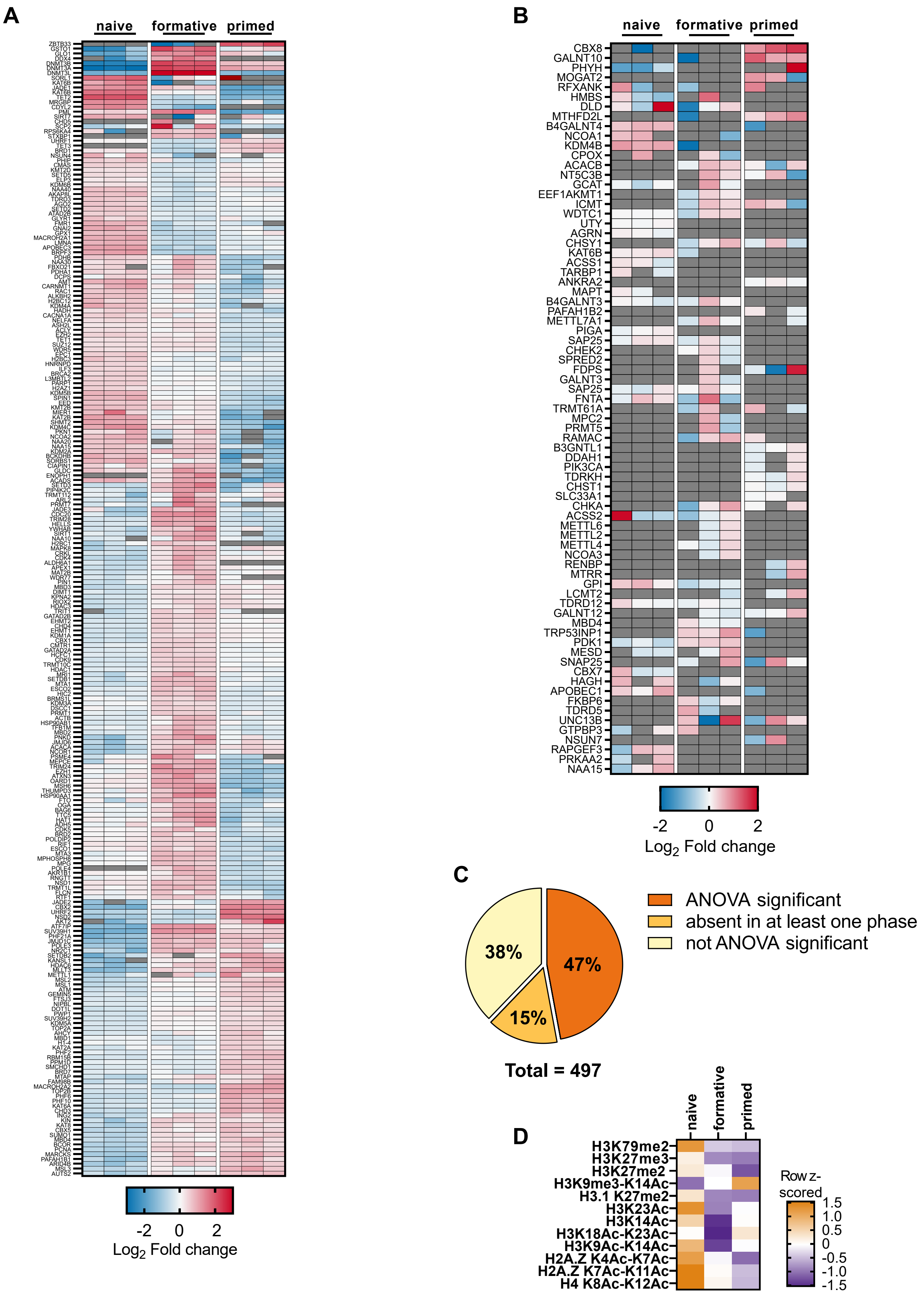

Figure S7 (Related to Figure 3)

Zinc finger (domain containing) proteins

A) ANOVA significant (FDR < 0.05, fold change difference ≥ 2)

B) Absent in at least one pluripotency phase

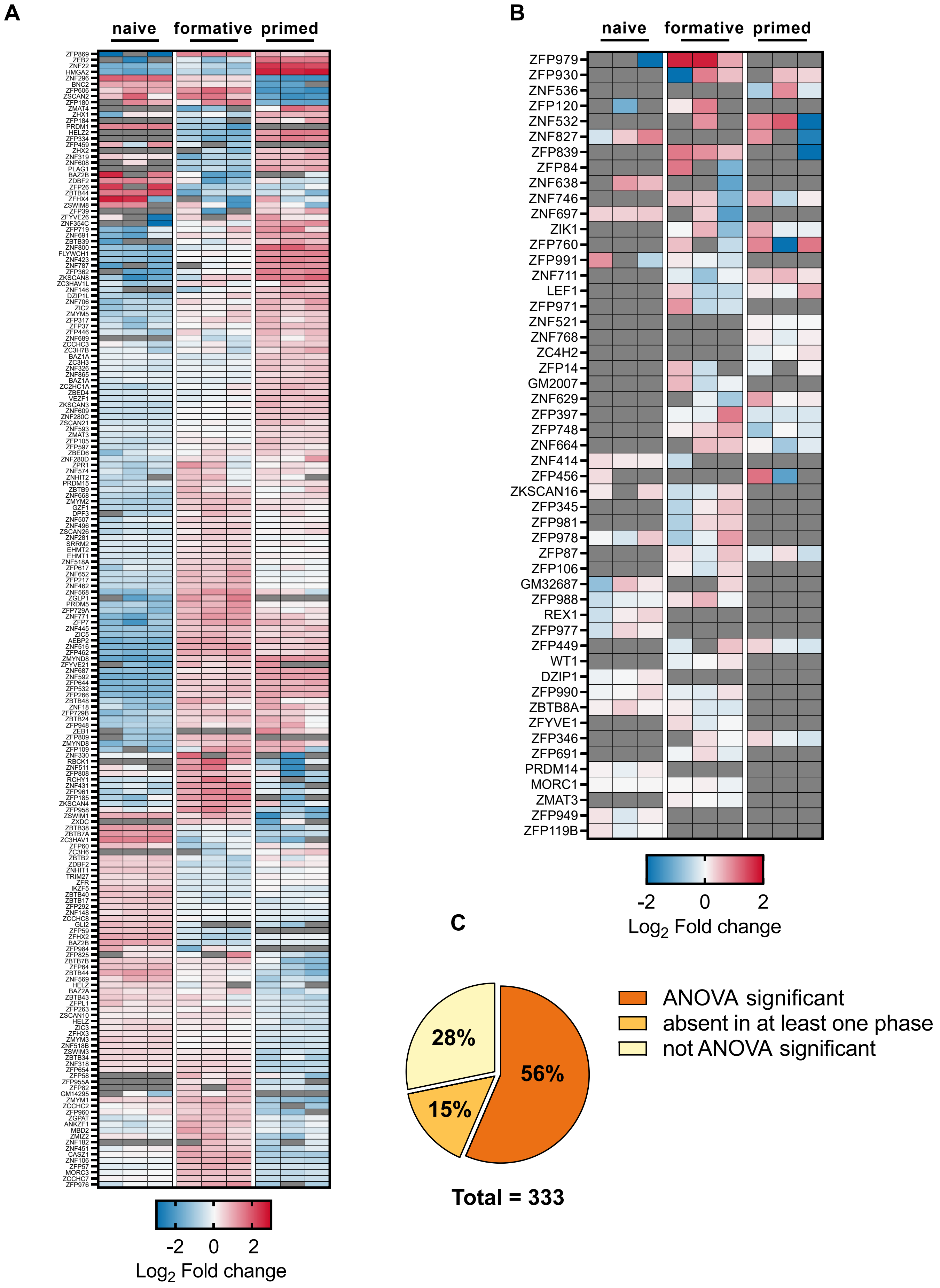

Figure S8 (Related to Figure 3)

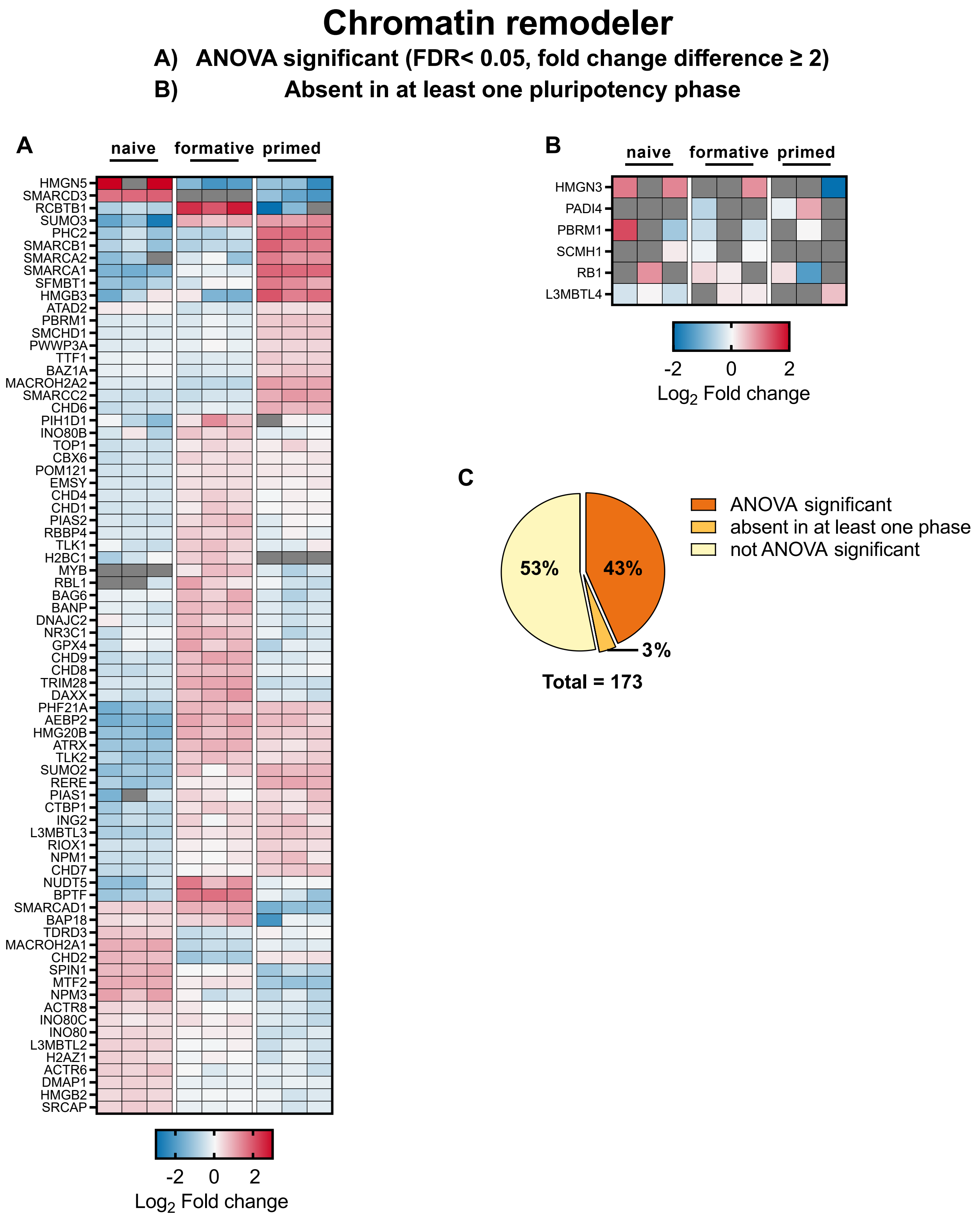

Figure S9 (Related to Figure 3)

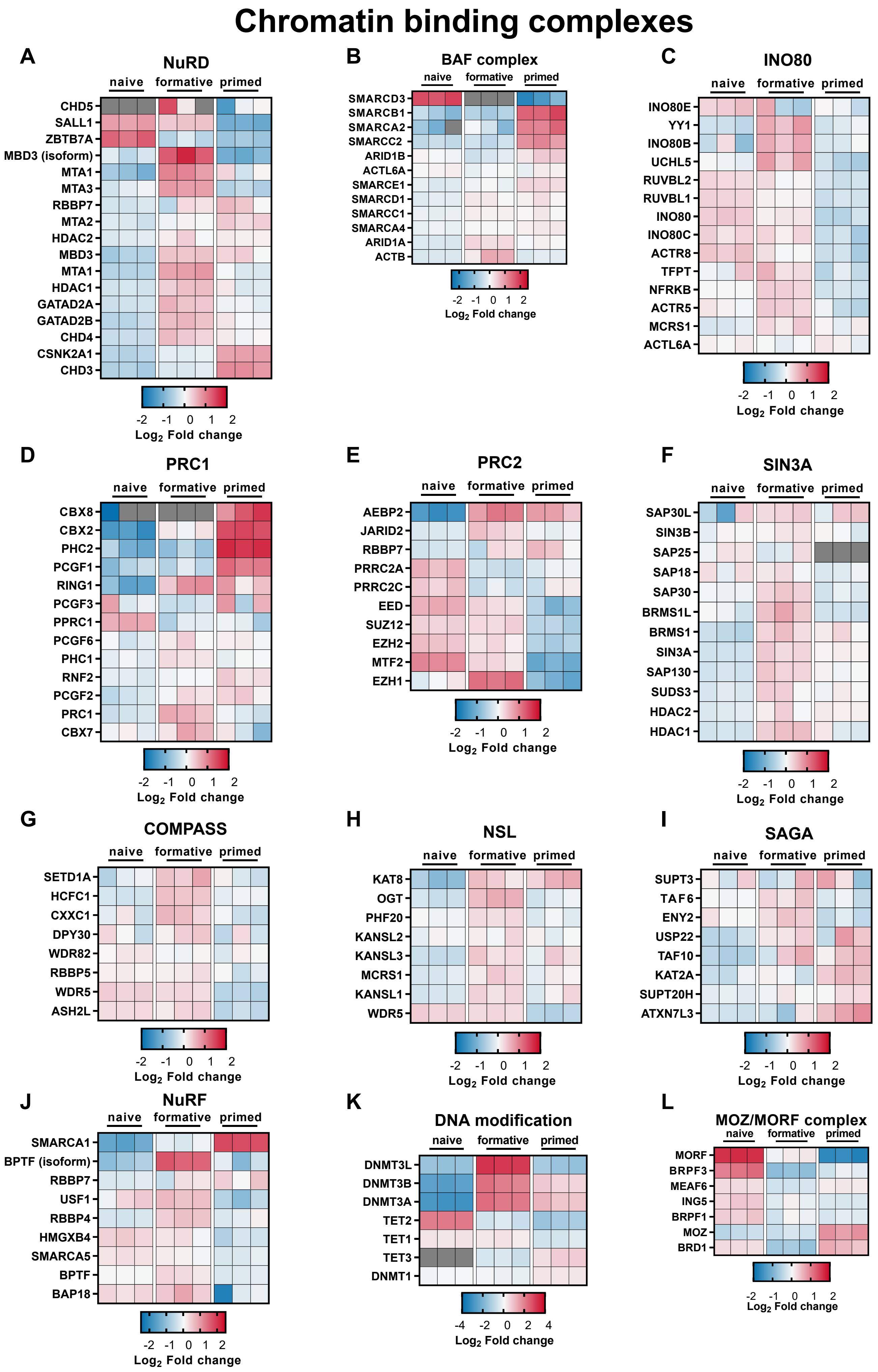

Figure S10 (Related to Figure 3)

Interactive Web Application:

A

https://pluripotency.shinyapps.io/Chromatome_Atlas/

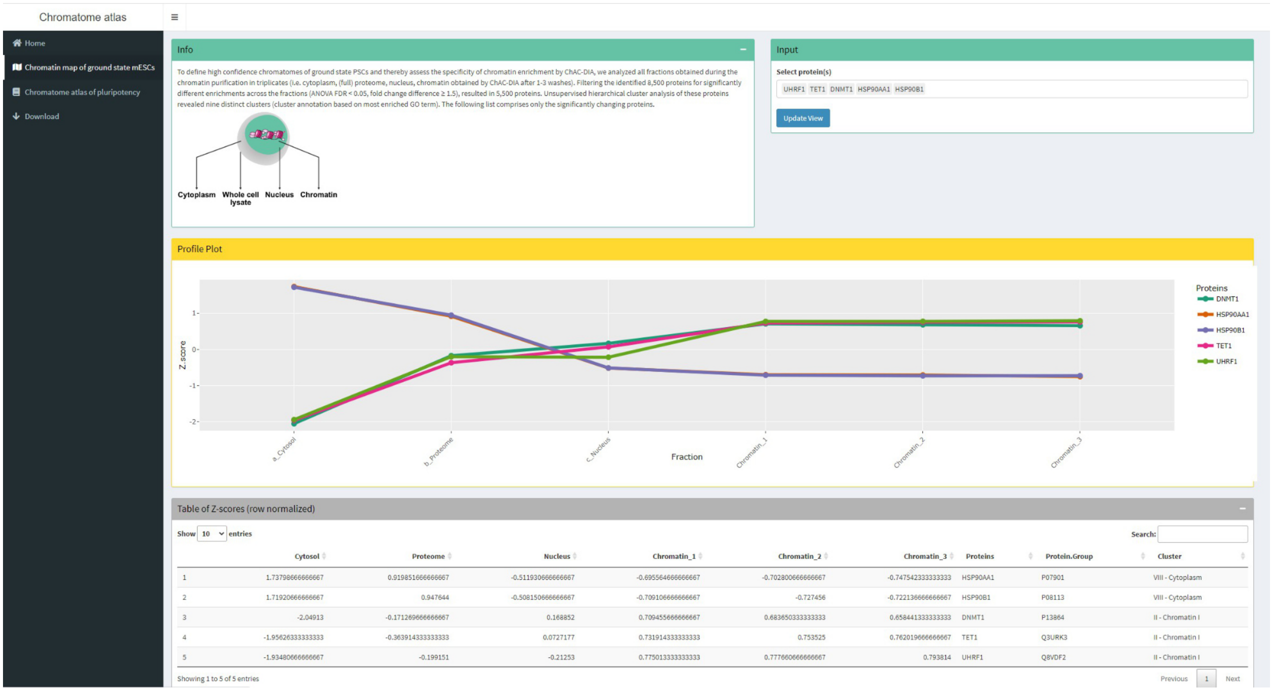

B

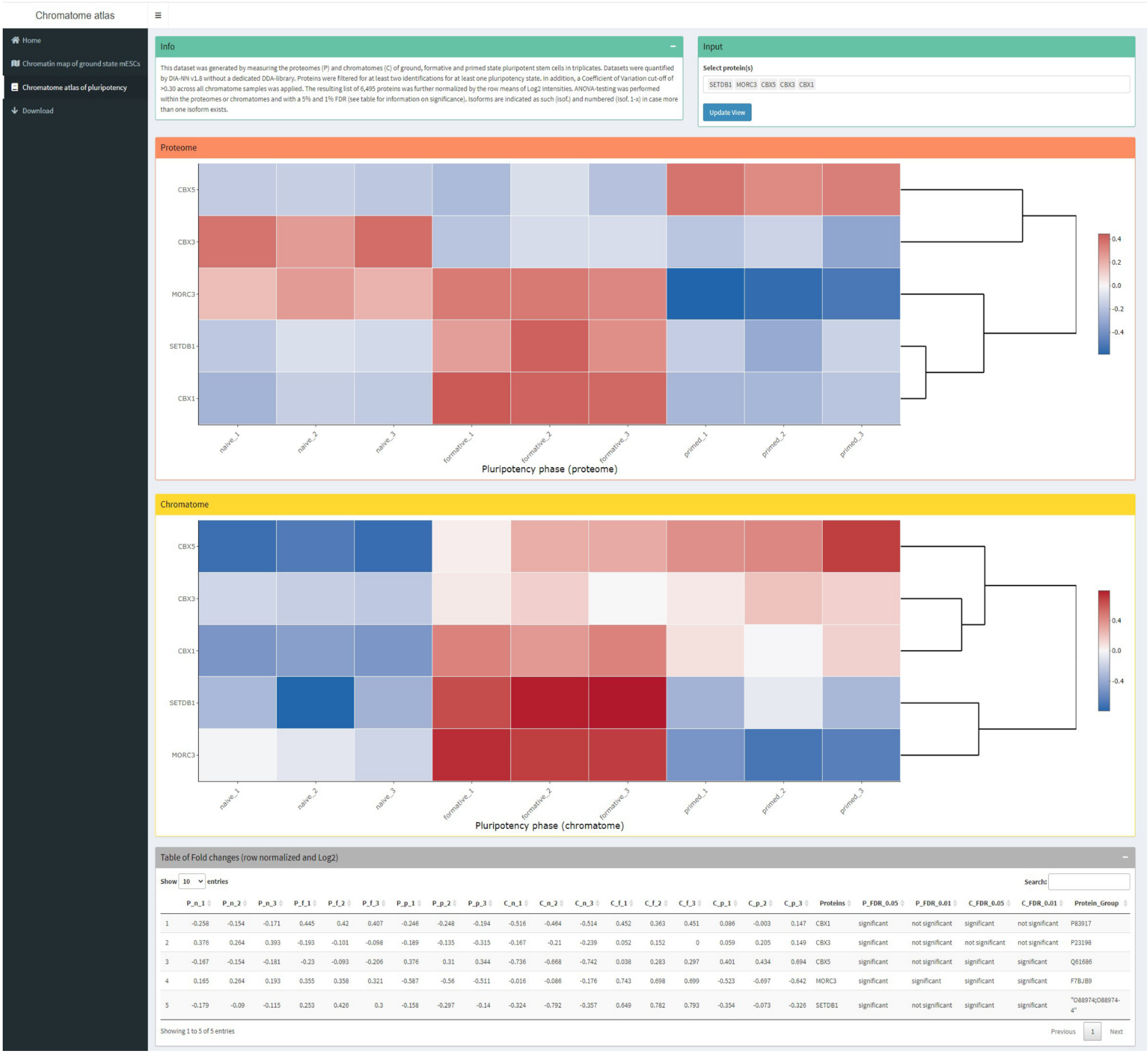

Figure S11 (Related to Figure 4)

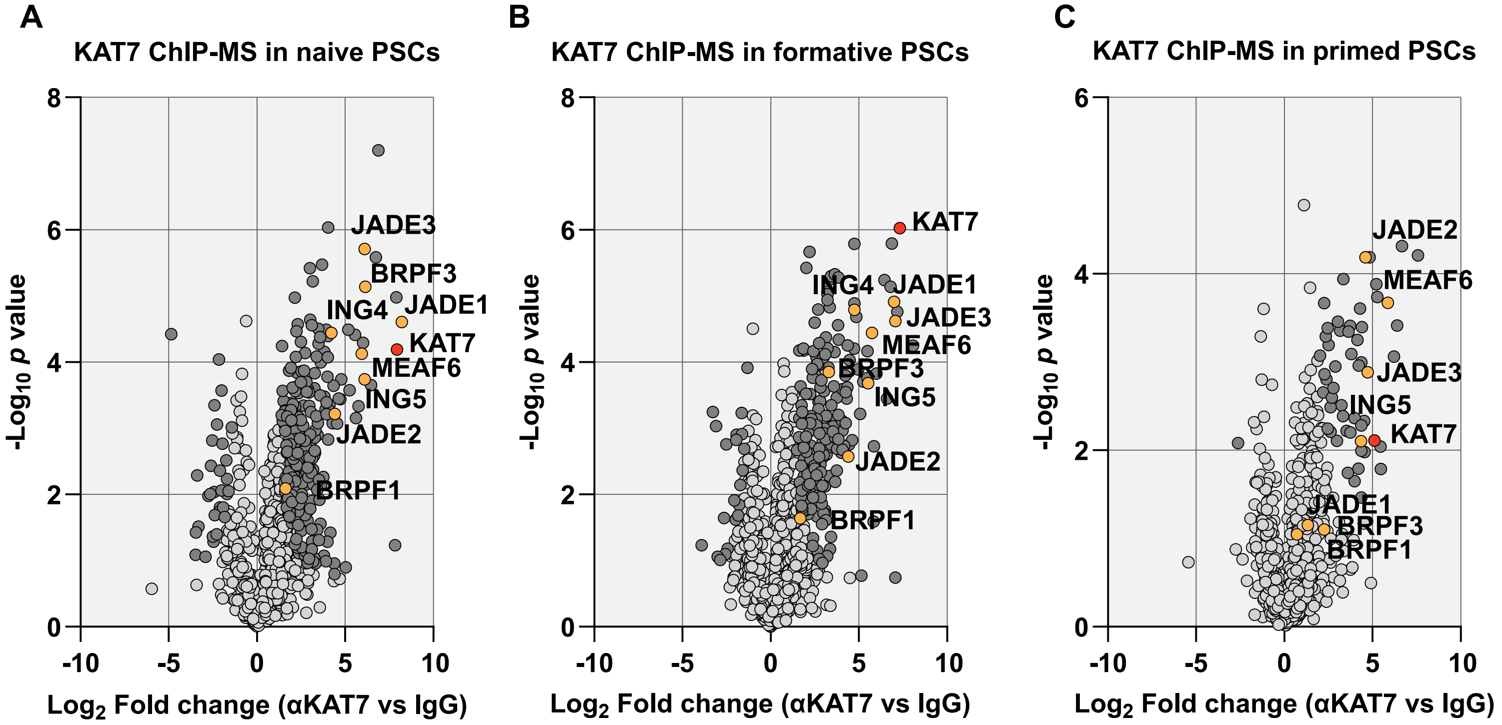

Figure S12 (Related to Figure 5)

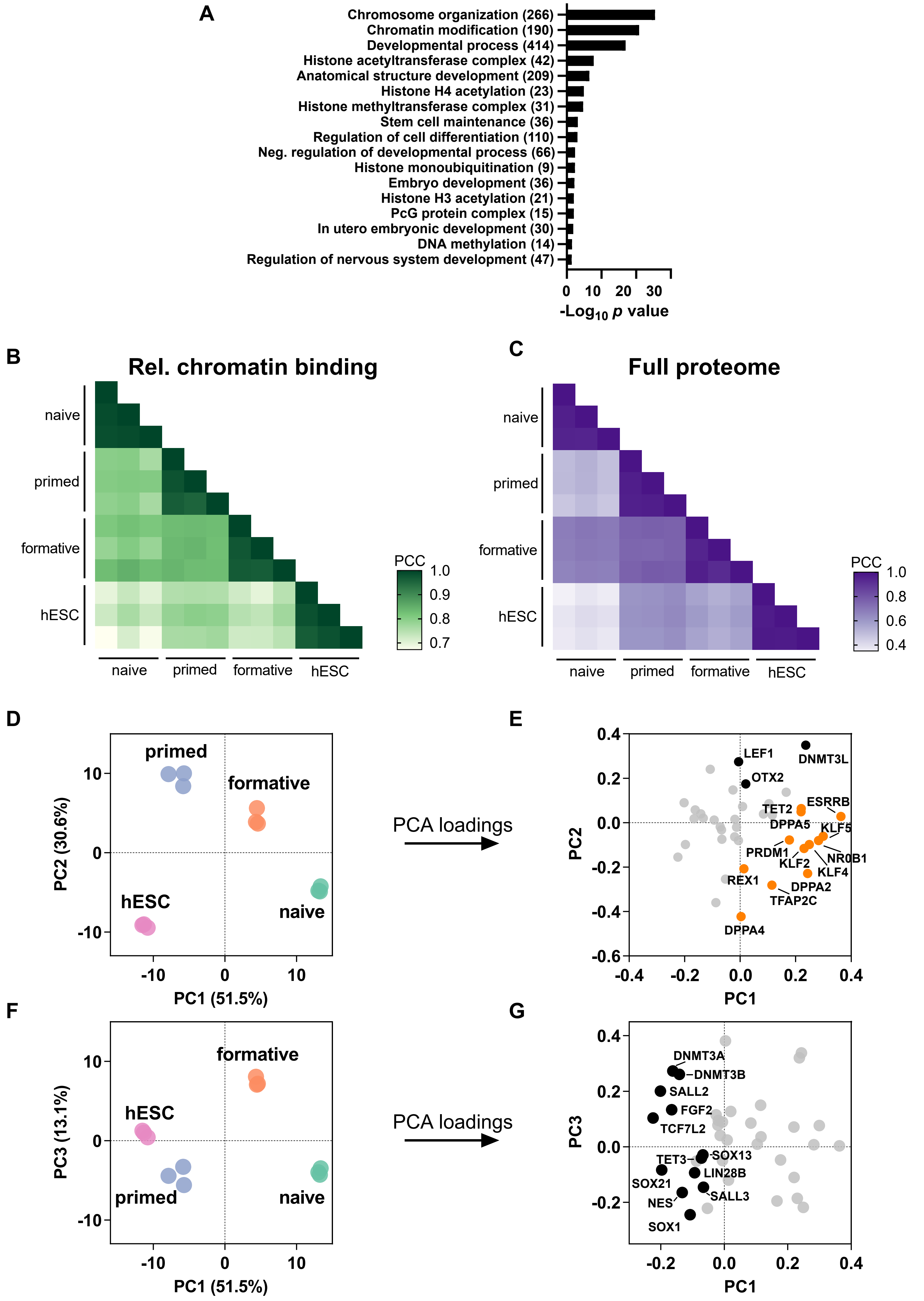

Supplemental Figure legends

Figure S1. Evaluation of ChAC-DIA improvements, Related to Figure 1

A, B Total numbers of identified proteins (**A**) or precursors (**B**) with representation of the percentage coefficient of variation (CV) below 20% and 10%. We performed our protocol with changing only one parameter at once. DDA: same ChAC workflow, but without DIA. DIA: PAC step is omitted (instead, acetone precipitation is performed). +RNase: additional incubation of nuclei with RNaseA for 15 min at 37°C. XL of whole cells: FA crosslinking is performed before nuclei isolation. FA+DSSO: double crosslinking with FA and DSSO. The data was analyzed with Spectronaut. Experimental conditions were kept comparable by using the same cell pool. Briefly, DIA improved the protein identification rate by 37.9% with constant CVs around 7.3% compared to just DDA. Despite more than doubling precursor numbers, CVs on peptide-level were even reduced from 15.6% for DDA to 12.7% for DIA. Strikingly, CVs were further improved by the additional PAC step (median CVs for proteins 4.1% and for precursors 9.6%). Moreover, additional RNAse addition prior to nuclei lysis and the formaldehyde crosslinking of whole cells instead of nuclei impaired CVs especially on precursor level (22.2% and 18.4%, respectively). A combination of DIA and PAC after nuclei isolation without RNase addition therefore gave the best results in terms of sensitivity and reproducibility. **C** Effect of precursor isolation window numbers in DIA on total precursor identifications and CV. The data was analyzed with DIA-NN. MS2 resolution was constant at 30,000. Here, we found that 30 or 40 isolation windows outperform 15 windows regarding total precursor identifications by 11.8% and 19.3%, respectively, while keeping the CVs constant. **D, E** Total numbers of identified proteins (**D**) or precursors (**E**) with representation of the percentage CVs below 20% and 10% (corresponding experiment to **Figure 1B**). We compare a previous Chromatin Enrichment for Proteomics (ChEP)-based study (PRIDE: PXD011782) to ChAC-DIA quantified in directDIA mode by either Spectronaut or DIA-NN with or without matching across different purification fractions which are mentioned in **Figure 2A**. **F, G** Total numbers of proteins (**F**) or precursors (**G**) falling into a gene ontology (GO) category (corresponding experiment to **Figure 1C**). **H** Distribution of CVs of proteins selected by GO category. Asterisks represent digits after the decimal point of 0.05. For better readability only comparisons on protein-level are shown. However, each comparison between PXD011782 and any given ChAC-DIA analysis method was to the same extent significant on precursor-level.

Figure S2. Comparison of chromatome to proteome and transcriptome, Related to Figure 1

A Venn diagram of proteins identified in proteome and chromatome of naive PSCs. Biological replicates: n = 3. **B** Numbers of proteins falling into a GO category and that are either identified in the proteome and chromatome or in one of the experiments exclusively. **C** Numbers of proteins falling into a GO category in the proteome or transcriptome of naïve PSCs. **D** Protein abundance rank based on the naive PSC proteome. Chromatin binding and DNA-binding proteins are highlighted in magenta. Displayed protein names indicate the highest ranked 9 proteins.

E Scatter plot of all mESC expressed mRNAs corresponding to annotated "chromatin-binding" binding proteins. Abundances of mRNAs/proteins are scaled from 0 to 1. Total proteome abundances are used to color each individual mRNA/protein. Proteome and chromatome raw files were only matched within technical triplicates using DIA-NN v1.8. **F** Analysis of expressed mRNAs corresponding to "chromatin binding" proteins which have additional "nuclear", "cytoplasmic" or "membrane" localization. The bar diagram represents their % among all proteins missed (violet) or identified (grey) by ChAC-DIA. **G** Abundances of all expressed mRNAs corresponding to "chromatin binding" proteins across different ChAC-DIA purification steps. MS raw files were analyzed together with matching between runs by DIA-NN v1.8.

Figure S3. Reproducibility of differential fraction analysis during ChAC-DIA, Related to Figure 2

A-D Unsuprevised hierarchical clustering of R^2 values (**A**) and scatter plots of nucleus vs ChAC-DIA (3x washes) (**B**), ChAC-DIA 1x wash vs 3x washes (**C**) and two replicates of ChAC-DIA (3x washes) (**D**). **E** Fisher's exact test to assess enriched GO terms of significantly enriched proteins in each cluster based on unsupervised hierarchical clustering in **Figure 2A**. P values are colour coded (-Log_{10}) and dot diameters correspond to group sizes (Log_2). **F** Percentage of a given GO category from the total cluster size of each cluster.

Figure S4. Complete chromatome list of pluripotency or differentiation related proteins, Related to Figure 3

A-C Heatmap representation of Log_2 FCs for significant differences between pluripotency phases (**A**), proteins absent in at least one pluripotency phase (**B**) or no ANOVA-based significant differences (**C**). **D** Total group size and percentage of (non-)significant differences.

Figure S5. Complete chromatome list of proteins annotated with „Transcription factor activity", Related to Figure 3

A, B Heatmap representation of Log_2 FCs for significant differences between pluripotency phases (**A**) or proteins absent in at least one pluripotency phase (**B**). **C** Total group size and percentage of (non-)significant differences.

Figure S6. Complete chromatome list of proteins related to epigenetic regulation, Related to Figure 3

A, B Heatmap representation of Log_2 FCs for significant differences between pluripotency phases (**A**) or proteins absent in at least one pluripotency phase (**B**). **C** Total group size and percentage of (non-)significant differences. **D** Selection of identified and ANOVA-significant histone posttranslational modifications (PTMs) identified by ChAC-DIA. Data was analyzed by Spectronaut, column-wise normalized to median column intensity and subsequently row z-scored and averaged across triplicates.

Figure S7. Complete chromatome list harboring a Zinc finger domain, Related to Figure 3

A, B Heatmap representation of Log_2 FCs for significant differences between pluripotency phases (**A**) or proteins absent in at least one pluripotency phase (**B**). **C** Total group size and percentage of (non-)significant differences.

Figure S8. Complete chromatome list of proteins annotated with „chromatin remodeler“ or „chromatin organization“, Related to Figure 3

A, B Heatmap representation of Log_2 FCs for significant differences between pluripotency phases (**A**) or proteins absent in at least one pluripotency phase (**B**). **C** Total group size and percentage of (non-)significant differences.

Figure S9. Examples of chromatin-associated complexes, Related to Figure 3.

A-L Heatmap representation of chromatin-associated complexes and their Log_2 FCs between pluripotency phases.

Figure S10. Interactive web application with example input, Related to Figure 3.

A Chromatin map of ground state mESCs. Profile plot shows relative abundance of proteins of interest in the cytoplasmic, full proteome, nuclear and chromatome (after 1-3 washes) fractions **B** Chromatome atlas of pluripotency. Users can search for proteins of interest and their respective full proteome and chromatome levels. Unlike for heatmaps shown in Supplementary Figures S4-S9, missing values were imputed.

Figure S11. ChIP-MS of KAT7 in naive, formative and primed PSCs, Related to Figure 4.

A-C Volcano plots of KAT7 (Abcam, ab70183) vs Normal Rabbit IgG control (Cell Signaling, 2729) ChIP-MS in WT mouse PSCs at naive, formative and primed pluripotency states (n = 3 independent replicates, except for primed IgG control, which had 2 independent replicates). Dark grey dots: significantly enriched proteins after KAT7 pulldown. Red dot: KAT7. Orange dots: Proteins associated with HBO1 complex. Light grey dots: not significantly enriched proteins. Statistical significance is based on a Student's t-test with a permutation-based FDR of 0.05 and an s_0-cutoff of >1 (based on Log_2 FCs). Represented Log_2 FCs are computed by using LFQ values and are not normalized for KAT7-levels, unlike **Figure 4L**, which shows KAT7-normalized iBAQ values.

Figure S12. Comparison of proteomes and chromatomes between mouse naive, formative and primed PSCs as well as hESCs, Related to Figure 6

A Fisher’s exact results obtained from comparing the shared high confidence chromatome across all four tested cell lines against the total set of high confidence chromatome binders. Numbers in brackets represent groups size. **B, C** Pearson correlations of relative chromatin binding (**B**) or full proteomes (**C**) filtered for pluripotency or differentiation markers as in **Figure 6C**. Underlying data was filtered for only valid values (**B**) or at least 6 valid values in total and missing values were imputed based on a gaussian distribution relative to the standard deviations of measured values (width of 0.2 and a downshift of 1.8 standard deviations) (**C**). **D-G** PCA representations of projections (**D, F**) and individual loadings (**E, G**) based on chromatome values of bona fide pluripotency and differentiation markers as represented in **Figure 6G** and from each mouse PSC and hESCs. **D, F** are based on PC1 and PC2 whereas **E, G** on PC1 and PC3. Orange dots represent pre- implantation markers and black dots post-implantation markers.
