# Supplementary material for: Comprehensive chromatin proteomics resolves functional phases of pluripotency and identifies changes in regulatory components
Source: Nucleic Acids Res. 2023 Feb 20;51(6):2671–90. doi: 10.1093/nar/gkad058 (PMC10085704; doi:10.1093/nar/gkad058)
Supplement: gkad058_Supplemental_Files [file gkad058_supplemental_files.zip › Supplementary Table Infomation.pdf]

Supplementary Table Infomation

Supplementary Table 1. Benchmarking data of Chromatin Aggregation Capture (ChAC) followed by data-independent MS acquisition (DIA), Related to Figure 1B-F, Supplementary Figure S1A-H and S2A-D

Supplementary Table 2. Chromatome map of naive mESCs based on ChAC-DIA, Related to Figure 2A-C and Supplementary Figure S3 AF

Supplementary Table 3. Chromatome atlas of mouse pluripotency phases, Related to Figure 3B-D, 4E,F, and Supplementary Figure S4-S11

Supplementary Table 4. Comparison of human and mouse pluripotency, Related to Figure 6A-I and Supplementary Figure S12A-G
